# Implementation of HER2DX Scores into Treatment Decisions in Early-Stage HER2-Positive Breast Cancer

**DOI:** 10.3390/ijms27125293

**Published:** 2026-06-11

**Authors:** Lola R. Nogueira, Ana Maderuelo, Manuel Alva Bianchi, Pablo Tolosa, Laura Lema, Juan Montes, Teresa Zumárraga, Ainhoa Madariaga, Luis Manso, Sofía Aragón Sánchez, Consuelo Sanz, Yolanda Ruano, Eva Ciruelos Gil, Rodrigo Sánchez-Bayona

**Affiliations:** 1 Servicio de Oncología Médica, Hospital Universitario 12 de Octubre, 28041 Madrid, Spain; 2 Instituto de Investigación Sanitaria Hospital 12 de Octubre (imas12), 28041 Madrid, Spain; 3 Servicio de Ginecología, Hospital Universitario 12 de Octubre, 28041 Madrid, Spain; 4 Servicio de Anatomía Patológica, Hospital Universitario 12 de Octubre, 28041 Madrid, Spain

**Keywords:** HER2-positive breast cancer, HER2DX, genomic assay, treatment de-escalation, neoadjuvant therapy, risk stratification, multidisciplinary decision-making

## Abstract

HER2DX is a 27-gene genomic assay generating three complementary scores: a pCR score predicting the likelihood of achieving pathological complete response (pCR, defined as absence of residual invasive disease after neoadjuvant therapy), a Risk score estimating long-term recurrence risk, and an *ERBB2* mRNA score quantifying HER2 transcriptional activation. Together, these scores may guide treatment escalation or de-escalation strategies in routine practice. We performed a single-center observational study at 12 de Octubre University Hospital (Madrid, Spain), including patients with early-stage HER2-positive breast cancer who underwent HER2DX testing (2023–2025), and analyzed clinicopathologic features, treatment decisions, and pathological outcomes. Forty-five patients were included (median age 57 years). Stage II accounted for 71.1% of cases; 66.7% were hormone receptor-positive, and 31.3% were node-positive. HER2DX modified clinical management in 51.1% of patients. The pCR score changed the initial strategy (neoadjuvant therapy versus upfront surgery) in 17.8% of cases and, independently, favored de-escalation to taxane plus dual HER2 blockade, with 90% of high-score tumors achieving a pathological complete response. The Risk score informed chemotherapy backbone selection within stage II disease. The *ERBB2* score correlated with HER2 immunohistochemical intensity. In this exploratory real-world cohort, HER2DX provided biologically distinct information beyond traditional immunohistochemistry and was associated with modifications in multidisciplinary treatment decision-making in early-stage HER2-positive breast cancer, warranting prospective validation in larger cohorts.

## 1. Introduction

HER2-positive breast cancer (BC) accounts for approximately 15–20% of all breast cancer cases and constitutes a distinct biological subtype characterized by HER2 overexpression and/or gene amplification, historically associated with aggressive clinical behavior and poor outcomes in the absence of targeted therapy [1]. The incorporation of anti-HER2 agents into preoperative chemotherapy regimens has dramatically improved survival outcomes in early breast cancer (EBC), transforming the natural history of this subtype and establishing dual HER2 blockade-based strategies as a standard of care [2,3]. In a setting historically dominated by anthracycline-based polychemotherapy, the introduction of dual HER2 blockade was the first advance in the therapeutic landscape that enabled the use of alternative chemotherapy backbones, including anthracycline-free regimens with or without platinum agents [4,5,6]. The recent clinical incorporation of antibody–drug conjugates into the HER2-positive treatment landscape further underscores the growing complexity of therapeutic strategies and the need to refine patient selection for escalation and de-escalation approaches.

Despite these advances, HER2-positive BC remains a biologically heterogeneous entity, with substantial variability in intrinsic subtype, immune microenvironment, and sensitivity to systemic therapy [7]. Although achieving a pathological complete response (pCR) after neoadjuvant treatment (NAT) is associated with favorable long-term outcomes, not all patients achieve equal benefit from standard NAT, exposing the possibility of overtreatment in selected subgroups and the need for improved risk stratification and treatment tailoring [8]. Given this profound biological heterogeneity, there is an increasing rationale for therapeutic de-escalation strategies—such as the omission of anthracyclines or carboplatin—yet traditional clinical–pathological variables often fail to fully capture tumor biology and relapse risk.

HER2DX is a 27-gene transcriptomic classifier that combines immune, proliferative, luminal, and *ERBB2* pathway signatures with clinical variables [9,10,11]. The assay generates three independent scores: a Risk score estimating long-term recurrence risk, a pCR score predicting the likelihood of achieving pCR following NAT, and an *ERBB2* score quantifying *ERBB2* mRNA expression and HER2-driven signaling. These scores have undergone rigorous analytical and clinical validation across independent perioperative cohorts in EBC [10,12,13]. The pCR score has demonstrated independent predictive value for pCR following trastuzumab–pertuzumab-based neoadjuvant therapy across multiple datasets, with pCR rates ranging from approximately 20% in the low-score group to over 74% in the high-score group [11,13], and has been shown to predict response independently of classical biomarkers such as HR (hormone receptor) status, TILs (tumor-infiltrating lymphocytes), and Ki-67 (OR 1.77; 95% CI 1.05–2.98; *p* = 0.030) [13]. The Risk score has shown independent prognostic significance for long-term recurrence-free survival irrespective of pathological response [10,12], and the *ERBB2* mRNA score has been associated with response to HER2-targeted neoadjuvant therapy and with benefit from de-escalated chemotherapy-free strategies in selected patients [14].

While previous studies have established the analytical validity and prognostic performance of its individual scores, less is known about the extent to which integration of HER2DX into routine clinical practice may influence therapeutic strategies, particularly regarding treatment escalation or de-escalation. In the present study, we evaluated whether conventional clinicopathologic variables could reproduce the biological and predictive stratification achieved by HER2DX scores and quantified the real-world impact of its implementation on therapeutic decision-making. Specifically, we assessed how the availability of the assay modified initial treatment strategies, including the selection of neoadjuvant therapy versus upfront surgery and the use or omission of polychemotherapy regimens. Our study provides real-world evidence for the integration of a genomic assay in the multidisciplinary care of patients with HER2-positive BC.

## 2. Results

### 2.1. Study Population and Clinical Outcomes

A total of 45 patients with stage I–II HER2-positive EBC were assessed. Median age was 57 years (range 33–82), and the predominant stage was II (71.1%). Tumors were mainly HR-positive (66.7%), invasive ductal carcinoma (86.7%), grade 3 (51.1%), and 31.3% presented with clinically node-positive disease. Baseline clinicopathological characteristics according to clinical stage are summarized in Table 1.

Beyond these baseline features, additional biological variables were explored. Sixteen tumors (35.6%) demonstrated strong HER2 protein overexpression (IHC 3+), whereas 29 cases (64.4%) were classified as IHC 2+ with confirmed gene amplification by FISH. Quantitative copy number data were available for 37 tumors ranging from 2.0 to 7.9 signals. Tumor-infiltrating lymphocytes (TILs) were assessed in pretreatment tumor samples, and, among 38 evaluable cases, the median level was 4% (mean 7.9%; 0–88%).

Regarding treatment patterns, overall, 32 patients (71.1%) received NAT, including 4/13 (30.8%) with stage I disease and 28/32 (87.5%) with stage II disease. All patients with stage I treated with NAT received taxane plus dual HER2 blockade (THP), whereas this regimen was used in only 61.5% (16/28) of patients with stage II disease. The remaining stage II patients received regimens containing carboplatin (CTHP) or anthracyclines (AC-THP). Breast-conserving surgery was performed in 36 patients (85.7%) and mastectomy in six patients (14.3%). Axillary management consisted of sentinel lymph node biopsy in all stage I patients and in 23 patients (79.3%) with stage II disease. For the remaining patients, a targeted axillary dissection was performed.

In terms of pathological outcomes, among patients receiving NAC, pCR was achieved in all stage I cases (4/4, 100%) and in 15 of 25 evaluable stage II cases (60%) who had undergone surgery at the time of analysis. Three stage II patients remained pending surgical resection and therefore are not included in this analysis.

### 2.2. HER2DX pCR Score

Overall, 21 patients (46.7%) were categorized as low pCR score, 12 patients (26.7%) as intermediate, and 12 patients (26.7%) as high pCR score. Stage I disease showed a numerically higher proportion of high pCR scores (38.5%), whereas stage II tumors were more frequently classified as low score (53.1%), although this distribution was not statistically significant (Fisher’s exact *p* = 0.3). pCR score distribution differed significantly according to HR expression. Among HR+/HER2+ tumors (n = 30), 20 patients (66.7%) were classified as low pCR score and only one (3.3%) as high score, whereas among HR-/HER2+ tumors (n = 15), 11 patients (73.3%) were classified as high score and only one (6.7%) as low score (OR 52.5, 95% CI 8.5–323.8, *p* < 0.01); given the small subgroup sizes, this estimate should be interpreted descriptively. pCR score also differed significantly according to IHC intensity, with high pCR scores observed in 8/16 IHC 3+ tumors (50.0%) versus 4/29 IHC 2+/FISH+ tumors (13.8%) (OR 6.7, 95% CI 1.9–23.1, *p* < 0.05). Patients with Ki-67 >30% were more frequently classified as high pCR score (11/23, 47.8%) compared with those with Ki-67 ≤30% (1/22, 4.5%) (OR 11.3, 95% CI 3.1–41.9, *p* < 0.05).

Overall, the pCR score modified the initial treatment strategy in 17.8% of the cohort. In stage I disease, 30.8% of patients were directed to NAT based on a high pCR score, whereas all low- and intermediate-score tumors underwent upfront surgery. In stage II disease, upfront surgery was performed in 12.5% of cases, exclusively in patients with low pCR scores and cN0 disease.

Among patients who received NAT, treatment intensity was influenced by pCR score category. Across high pCR score tumors, 10 of 11 patients (90.9%) received de-escalated NAT with THP, compared with only 52.6% of patients in the low- or intermediate-score tumors. Accordingly, pCR score was independently associated with de-escalation to THP (OR 2.84; 95%CI 1.1–7.5, *p* < 0.05), leading to modification of the NAT regimen in 31.3% of treated patients. Adjustment of NAT intensity was not associated with post-surgical residual cancer burden (RCB). After treatment modulation, pCR was achieved in 90.0% of the high-score group (9/10 evaluable patients), 85.7% (6/7) of the intermediate-score group, and 33.3% (4/12) of the low-score group (Fisher’s exact *p* < 0.05), suggesting that HER2DX-guided treatment modulation did not adversely affect pathological outcomes in this cohort.

### 2.3. HER2DX Risk Score

Among the 45 patients, 30 (66.7%) were classified as low-risk and 15 (33.3%) as high-risk, with significant differences among clinical stages (*p* < 0.05). Across stage I tumors, 92.3% were low-risk, whereas only 56.3% of stage II tumors were low-risk. In contrast, Risk score distribution was not found to be associated with classical biomarkers according to tumor phenotype (HR+/HER2+ vs. HR-/HER2+) (χ^2^ *p* = 0.5). Similarly, no association was observed between the Risk score and HR expression, TILs, or Ki-67.

Despite its association with stage, the Risk score did not influence the choice between upfront surgery and NAT, with similar neoadjuvant rates among stage II high- and low-risk tumors (88.9% vs. 85.7%), whereas in stage I, only one of 13 tumors (7.7%) was high-risk, limiting subgroup comparisons. However, it did influence regimen selection among the NAT subgroup. Anthracycline-based therapy was administered exclusively in the high-risk group (18.2%) and was not used in any low-risk tumor. Conversely, anthracycline-free regimens were used more frequently in low-risk cases (78.9%) than in high-risk ones (45.5%), although this trend did not reach statistical significance (*p* = 0.16). Despite treatment adjustment, RCB was again similar across subgroups.

Clinicopathologic associations, treatment decisions, and pathological outcomes for both pCR and Risk scores are summarized in Table 2.

### 2.4. HER2DX ERBB2 mRNA Score

*ERBB2* mRNA score did not correlate with clinical stage, with comparable proportions of high-score tumors in stage I and stage II disease (61.5% vs. 62.5%). *ERBB2* score was not significantly associated with HR expression (OR 2.2, 95% CI 0.6–8.5), but was strongly related to HER2 IHC intensity, with high *ERBB2* scores observed in 15/16 IHC 3+ tumors (93.8%) versus 13/29 IHC 2+/FISH+ tumors (44.8%) (OR 19.2, 95% CI 2.2–164.3, *p* < 0.05). Within the neoadjuvant setting, *ERBB2* score further guided treatment regimen selection, as higher *ERBB2* scores were more frequently managed without anthracyclines (OR 4.5, 95% CI 1.3–15.7, *p* < 0.05), favoring taxane-based dual HER2 blockade regimens with reduced chemotherapy burden. In the adjuvant setting, no clear association was observed between treatment and any of the scores, although the limited number of stage II patients treated upfront precluded meaningful subgroup analysis.

**Figure 1:**
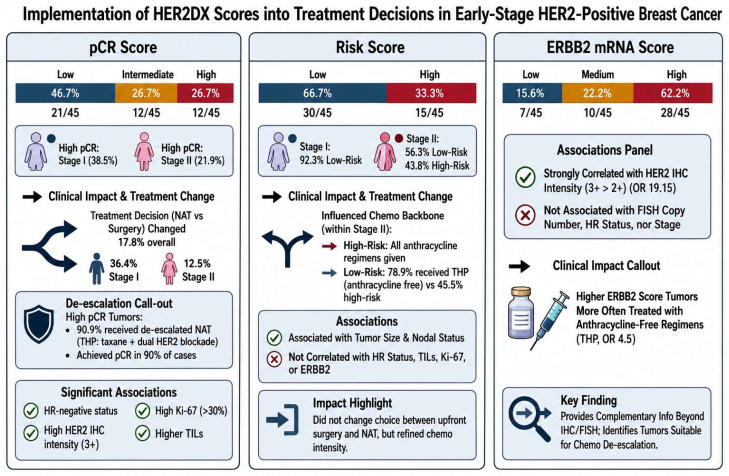
Distribution and clinical implications of HER2DX scores across clinical stages. Results of the three HER2DX scores according to clinical stage—pCR score (**left**), Risk score (**center**), and *ERBB2* score (**right**)—highlight their main clinicopathologic associations and their impact on treatment decisions within the cohort.

Overall, the distribution of HER2DX scores and their key clinicopathologic associations and clinical implications are summarized in Figure 1.

### 2.5. Association of HER2DX Scores and HER2 Gene Copy Number

Interestingly, none of the HER2DX scores were associated with HER2 gene copy number in the FISH-tested subset (pCR score: OR 1.00, 95% CI 0.29–3.39; Risk score: OR 1.05, 95% CI 0.28–3.92), although a non-significant trend was observed for the *ERBB2* score (OR 3.37, 95% CI 0.92–12.33), noting that FISH was predominantly performed in tumors with equivocal (IHC 2+) HER2 expression.

Also, none of the HER2DX scores showed a significant correlation with HER2 gene copy number in the FISH-tested subset (Spearman’s rho 0.24 for pCR score, 0.07 for Risk score, and 0.24 for *ERBB2* score; all *p* > 0.20). These associations are illustrated in Supplementary Figure S1.

## 3. Discussion

In our real-world cohort, HER2DX scores were associated with modifications in therapeutic decision-making in 51.1% of patients by guiding both the indication for NAT and adjustments in regimen intensity. The pCR score appeared to inform selection of NAT versus upfront surgery, with modification of the initial treatment strategy observed in 17.8% of patients, with a greater impact in stage I disease with high pCR scores, whereas in stage II disease, upfront surgery was selected in low pCR, cN0 patients. Beyond treatment indication, HER2DX also appeared to inform chemotherapy intensity, consistent with modification of the NAT regimen in 31.3% of treated patients. This effect was driven by the complementary contribution of its components: the pCR score supported de-escalation in tumors with high probability of response, while the Risk score showed a trend toward further stratifying treatment intensity, with a ≈30% greater use of anthracycline-free regimens in low-risk tumors and anthracyclines administered exclusively in the high-risk subgroup, with the *ERBB2* score providing additional support for anthracycline-sparing strategies.

**Figure 2:**
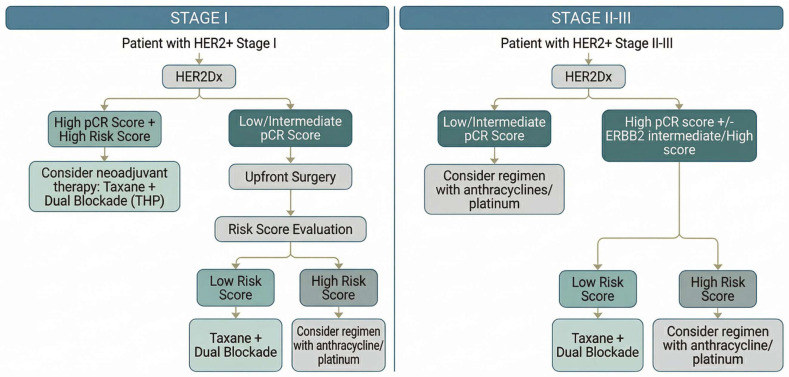
Proposed treatment decision algorithm integrating HER2DX scores in HER2-positive EBC. In stage I disease, the pCR and Risk scores help to identify patients who may benefit from neoadjuvant therapy, whereas tumors with low or intermediate scores may proceed to upfront surgery followed by risk-adapted systemic treatment. In stage II–III disease, the pCR and *ERBB2* scores inform the likelihood of response to HER2-targeted therapy and may support de-escalation to anthracycline-sparing adjuvant regimens in biologically favorable tumors. The Risk score further refines treatment intensity by identifying patients who may require more aggressive chemotherapy backbones. This algorithm represents a proposed workflow derived from a small single-center experience. It is not a validated clinical guideline and should not be used to guide individual treatment decisions outside of a prospective research context. The proposed framework extends beyond the present cohort and reflects the broader clinical application of HER2DX across stage I–III disease [10].

These findings can be translated into a stage-adapted clinical algorithm (Figure 2), illustrating how HER2DX components may guide treatment choice. This approach is particularly relevant in the current therapeutic landscape of HER2-positive EBC, where improved outcomes driven by anti-HER2 therapies have added complexity to clinical decision-making. Accordingly, the focus has shifted toward balancing efficacy with toxicity through treatment de-escalation, with HER2DX potentially providing a multidimensional assessment beyond conventional clinicopathologic factors, integrating relapse risk, probability of response, and *ERBB2* mRNA expression.

Biologically, HER2DX components capture distinct dimensions that are not adequately represented by conventional clinicopathological biomarkers. Achievement of pCR has emerged as a key endpoint in EBC, given its consistent association with improved long-term survival, irrespective of the NAT administered [11]. Within this framework, HER2DX has demonstrated the ability to predict pCR after trastuzumab–pertuzumab-based therapy [15]. In our study, higher pCR scores correlated with TIL levels, HR status, HER2 IHC, and ki-67.

The correlation between TIL levels and the pCR score reflects the immune-related gene signatures incorporated into this score of the HER2DX assay, including the 14-gene IgG signature [14,16]. While TILs provide a morphologic estimate of immune infiltration and have been associated with pCR [17], the IgG signature captures functional immune activation and has demonstrated even stronger predictive and prognostic value. In contrast, neither risk nor *ERBB2* scores showed significant correlations with TIL levels in our cohort, consistent with prior reports indicating that these components rely on distinct biological and clinical determinants and reinforcing the biological non-redundancy of HER2DX components [18].

The pCR score distribution was also found to differ significantly according to HR status, with higher pCR scores observed in HER2-positive/HR-negative tumors compared with HR-positive/HER2-positive tumors. Also, according to HER2 IHC, higher scores were strongly associated with 3+ HER2-overexpressing tumors. Biologically coherent, as HR-negative tumors are more likely to be predominantly driven by HER2-mediated oncogenic signaling, enhancing sensitivity to HER2-targeted therapy, whereas HR-positive tumors may retain parallel estrogen receptor pathway activation that attenuates exclusive HER2 dependency [19]. Similarly, strong HER2 IHC expression (IHC 3+) reflects robust activation of the HER2 pathway in contrast to lower HER2 intensity tumors that may rely on co-dominant or alternative signaling pathways [20]. Finally, the significant association between pCR score and Ki-67 in our cohort indicates that the score captures proliferative biology, aligning with evidence that highly proliferative tumors are more responsive to systemic treatment. The pCR rates by score group observed in our cohort (33.3%, 85.7%, and 90.0% for low, medium, and high, respectively) are consistent with those reported in the largest prospective real-world series to date (13.3%, 51.6%, and 81.8%; n = 297), in which HER2DX-guided de-escalation achieved comparable pCR rates to multi-agent chemotherapy in high-score patients (81.5% vs. 69.0%; OR 1.97; *p* = 0.26) [21], supporting the real-world feasibility of score-guided treatment personalization without compromising pathological outcomes.

In contrast to the pCR component, the Risk score reflects long-term recurrence biology rather than immediate treatment sensitivity. In our cohort, the Risk score distribution differed significantly according to both tumor size and nodal status. However, its correlation with TIL levels, HER2 IHC, or *ERBB2* score was not statistically significant, underscoring that relapse risk cannot be inferred solely from treatment sensitivity [10]. In our series, the incorporation of the Risk score contributed to the modulation of NAT intensity within the same clinical stage. This concept is currently being prospectively evaluated in the DEFINITIVE trial, which assigns treatment intensity according to HER2DX molecular risk in stage II–III disease [22]. Similarly, adaptive strategies such as PHERGain and the ongoing phase III PHERGain-2 trial explore biology-driven de-escalation approaches, although based on early metabolic response rather than baseline transcriptomic risk [23].

Beyond treatment sensitivity (pCR score) and long-term recurrence risk (Risk score), the *ERBB2* component adds a third and biologically distinct layer of information by quantifying HER2 pathway activation at the transcriptomic level. Importantly, HER2 amplification, HER2-enriched intrinsic subtype, and *ERBB2* mRNA levels represent related but non-equivalent biological constructs. In our cohort, the close relationship between HER2 immunohistochemical intensity and lack of association with FISH copy number in equivocal cases supports the concept that transcriptional output does not necessarily parallel genomic amplification. This observation is consistent with prior evidence demonstrating that *ERBB2* mRNA levels provide clinically meaningful information beyond conventional HER2 classification and intrinsic subtype assignment [13,18]. Therefore, the preferential use of anthracycline-sparing regimens in tumors with higher *ERBB2* scores in our series supports the hypothesis that strong HER2 transcriptional activation may identify tumors sufficiently dependent on HER2 signaling to allow treatment strategies centered on targeted blockade.

This study has some limitations to be acknowledged. Its single-center design and relatively small sample size should be acknowledged, although they allowed for a consistent clinical approach and detailed characterization of the cohort. Due to the limited size of some subgroups, these estimates should be interpreted with care, as confidence intervals remain wide and the risk of unstable effect size estimates is non-trivial. HER2DX testing was used in selected cases of clinical uncertainty—defined as situations where standard clinicopathologic variables were insufficient to determine the optimal treatment strategy—identified by multidisciplinary tumor board consensus, which may have influenced the generalizability of our findings to the broader population. Additionally, the absence of a contemporaneous non-HER2DX-tested control cohort precludes definitive causal conclusions regarding the impact of the assay on treatment decisions and clinical outcomes. Treatment modifications were defined relative to a retrospectively reconstructed stage- and guideline-based institutional standard. While this approach minimizes anchoring and desirability bias inherent to prospective physician self-report methods, residual confounding by unmeasured clinical factors cannot be excluded in an observational study of this design. Despite these limitations, the study reflects real-world clinical practice and provides meaningful insights into the implementation of HER2DX in routine decision-making.

## 4. Materials and Methods

We conducted a single-center observational study with patients diagnosed with early-stage HER2-positive breast cancer for whom a HER2DX genomic test was performed per clinical practice at the 12 de Octubre University Hospital (Madrid, Spain) between February 2023 and December 2025. Eligible patients had histologically confirmed invasive HER2-positive breast carcinoma and available HER2DX reports.

To specifically assess the clinical impact of HER2DX on treatment decision-making, we defined a priori the primary objective as the proportion of cases in which HER2DX results modified the initially planned therapeutic strategy. The primary endpoint was a composite measure including: (i) changes in the indication for neoadjuvant therapy versus upfront surgery, and (ii) modifications in the intensity of the chemotherapy regimen (e.g., de-escalation to anthracycline-free regimens). The baseline treatment plan was retrospectively reconstructed as the stage- and nodal status-adapted standard of care consistent with ESMO guidelines. This design minimizes potential bias inherent to pre-test/post-test physician-reported approaches (e.g., anchoring or desirability bias), as the comparator was a predefined institutional standard. Final treatment decisions were established through multidisciplinary tumor board discussion. Specifically, treatment modifications were defined as de-escalation strategies in low-risk patients (upfront surgery in stage II cN0 disease, or anthracycline- and platinum-free regimens) and escalation strategies in high-risk patients (neoadjuvant therapy in stage I disease). As per institutional protocol, all early breast cancer cases are discussed at the multidisciplinary tumor board. HER2DX testing was indicated in patients for whom the tumor board identified clinical uncertainty regarding treatment strategy. All cases meeting this criterion during the study period underwent testing and were further included. Secondary objectives included the evaluation of associations between HER2DX scores and pathological complete response (pCR), residual cancer burden (RCB), and type of systemic treatment administered.

Clinicopathological variables were collected from electronic medical records. HER2 status was determined according to international guidelines using IHC and, when indicated, dual-probe FISH (HER2/CEP17 ratio). Tumors with IHC 3+ were considered HER2-positive, while equivocal (2+) cases underwent confirmatory FISH testing, and quantitative HER2 copy number was recorded when performed. HER2 status was determined by immunohistochemistry (IHC) and/or fluorescence in situ hybridization (FISH) according to international ASCO/CAP guidelines [24]. *ERBB2* gene amplification was quantified by dual-probe FISH (HER2/CEP17). HER2DX testing was conducted in a centralized laboratory (Reveal Genomics, Barcelona, Spain) on formalin-fixed paraffin-embedded (FFPE) pretreatment core needle biopsy samples. This 27-gene assay quantifies tumor- and immune-related gene expression, integrating four biological signatures—immune infiltration, proliferation, luminal differentiation, and HER2 amplicon expression—to generate three predefined genomic scores: pCR (low/intermediate/high), Risk (low/high), and *ERBB2* (low/medium/high), categorized according to validated manufacturer cut-offs. Pathological response following neoadjuvant therapy was assessed using the RCB classification. Ki-67 was assessed as a continuous variable and dichotomized using a data-driven cut-off of 30%, corresponding to the median value of the study cohort. TILs were assessed on hematoxylin and eosin-stained tumor sections and quantified as the percentage of stromal area occupied by mononuclear inflammatory cells, according to international recommendations, and reported as a continuous variable (0–100%).

All statistical analyses were performed using Stata/BE version 18 (StataCorp, College Station, TX, USA). Associations between categorical variables were evaluated using Pearson’s χ^2^ test or Fisher’s exact test when appropriate. Ordinal associations were assessed using Spearman’s rank correlation coefficients (ρ). Comparisons of ordinal or non-normally distributed variables between groups were performed using the Wilcoxon rank-sum test. To control for potential confounding, multivariable logistic and ordered logistic regression models were constructed when clinically appropriate, adjusting for clinical stage and other relevant covariates. Given the observed association between Risk score and stage, stratified analyses by stage were performed in selected comparisons to minimize confounding effects. Interaction terms between HER2DX scores and treatment intensity were explored to assess potential effect modification. All statistical tests were two-sided, and a *p*-value < 0.05 was considered statistically significant.

The datasets generated and analyzed during the current study are available from the corresponding author upon reasonable request. Data are not publicly available due to institutional and privacy restrictions.

## 5. Conclusions

In this exploratory real-world cohort of early-stage HER2-positive breast cancer, HER2DX informed conventional clinicopathologic assessment and was associated with modifications in therapeutic decision-making. These findings suggest that genomic information from HER2DX was associated with treatment modifications in routine multidisciplinary practice, without an observed detrimental effect on pathological response rates in this exploratory cohort, warranting prospective validation in larger series.

## Figures and Tables

**Table 1:** Baseline clinicopathological characteristics stratified by clinical stage.

	Stage I% (n)	Stage II% (n)	nN0% (n)	nN1% (n)
n	28.8% (13)	71.1% (32)	77.8% (35)	22.2% (10)
Age	55.5 (36.2–76.6)	58.4 (32.9–81.9)	56.8 (36.2–81.9)	55.2 (32.9–70.7)
T (mm)	12 (4–18)	24 (8–100)	22.1 (4–60)	30 (8–100)
Histology				
Ductal	84.6% (11)	87.5% (28)	85.7% (30)	90% (9)
Lobulillar	15.38% (2)	9.38% (3)	11.4% (4)	10% (1)
NOS	0	2.1% (1)	2.9% (1)	0
Grade				
1	0	3.2% (1)	0	10% (1)
2	46.6% (6)	48.4% (15)	52.9% (18)	30% (3)
3	53.9% (7)	48.4% (15)	47.1% (16)	60% (6)
PCR Score				
Low	30.8% (4)	53.1% (17)	48.6% (17)	40% (4)
Medium	30.8% (4)	25% (8)	28.6% (10)	20% (2)
High	38.5% (5)	21.9% (7)	22.9% (8)	20% (2)
Risk Score				
Low	92.31% (12)	56.3% (18)	80% (28)	20% (2)
High	7.7% (1)	43.8% (14)	20% (7)	80% (8)
*ERBB2* Score				
Low	15.4% (2)	15.6% (5)	17.1% (6)	10% (1)
Medium	23.1% (3)	21.9% (7)	22.9% (8)	20% (2)
High	61.5% (8)	62.5% (20)	60% (21)	70% (7)

NOS, not otherwise specified.

**Table 2:** HER2DX pCR score and Risk score: clinicopathologic associations and treatment impact.

	pCR Score				Risk Score		
	Low (n = 21)	Intermediate (n = 12)	High (n = 12)	*p*	Low (n = 30)	High (n = 15)	*p*
**A. Clinicopathologic associations**							
Clinical stage II, n (%)	17 (81.0)	8 (66.7)	7 (58.3)	0.30	18 (60.0)	14 (93.3)	<0.05
HR-positive, n (%)	19 (90.5)	6 (50.0)	1 (8.3)	<0.01	20 (66.7)	10 (66.7)	0.50
HER2 IHC 3+, n (%)	4 (19.0)	6 (50.0)	10 (83.3)	<0.05	11 (36.7)	5 (33.3)	0.82
Ki-67 > 30%, n (%)	6 (28.6)	7 (58.3)	10 (83.3)	<0.05	19 (63.3)	9 (60.0)	0.82
**B. NAT vs. upfront surgery**							
Stage I (n = 13)	4 (30.8)	4 (30.8)	5 (38.5)		12 (92.3)	1 (7.7)	
Upfront surgery, n/N (%)	4/4 (100)	4/4 (100)	1/5 (20)	<0.01	8/12 (66.7)	1/1 (100)	0.48
NAT, n/N (%)	0/4 (0)	0/4 (0)	4/5 (80)		4/12 (33.3)	0/1 (0)	
Stage II (n = 32)	17 (53.1)	8 (25.0)	7 (21.9)		18 (56.3)	14 (43.8)	
Upfront surgery, n/N (%)	4/17 (23.5)	0/8 (0)	0/7 (0)	0.147	2/18 (11.1)	3/14 (21.4)	0.79
NAT, n/N (%)	13/17(76.5)	8/8 (100)	7/7 (100)		16/18 (88.9)	12/14 (85.7)	
**C. NAT regimen intensity (among neoadjuvant patients)**							
THP, n/N (%)	6/11 (54.5)	4/8 (50.0)	10/11 (90.9)	<0.05	15/19 (78.9)	5/11 (45.5)	0.16
TCHP, n/N (%)	4/11 (36.4)	4/8 (50.0)	0/11 (0)		4/19 (21.1)	4/11 (36.4)	
AC-THP, n/N (%)	1/11 (9.1)	0/8 (0)	1/11 (9.1)		0/19 (0)	2/11 (18.2)	<0.05
**D. Pathological outcomes (among neoadjuvant patients)**							
pCR overall, n/N (%)	4/12 (33.3)	6/7 (85.7)	9/10 (90.0)	<0.05	12/19 (63.2)	7/10 (70.0)	NS
pCR, Stage I, n/N (%)	—	—	4/4 (100)	—	4/4 (100)	—	—
pCR, Stage II, n/N (%)	4/12 (33.3)	6/7 (85.7)	5/6 (83.3)	<0.05	8/15 (53.3)	7/10 (70.0)	NS

Abbreviations: HR, hormone receptor; IHC, immunohistochemistry; THP, taxane + trastuzumab + pertuzumab; TCHP, THP + carboplatin; AC-THP, anthracycline-containing regimen; NAT, neoadjuvant therapy; pCR, pathological complete response; NS, not significant. *p*-values from Fisher’s exact test unless otherwise stated. Results are exploratory given the small sample size.

## Data Availability

The data presented in this study are available on request from the corresponding author due to intellectual property.

## Supplementary Materials

The following supporting information can be downloaded at: https://www.mdpi.com/article/10.3390/ijms27125293/s1.
