# Supplementary material for: Implementation of HER2DX Scores into Treatment Decisions in Early-Stage HER2-Positive Breast Cancer
Source: Int J Mol Sci. 2026 Jun 11;27(12):5293. doi: 10.3390/ijms27125293 (PMC13300713; doi:10.3390/ijms27125293)
Supplement: Supplementary file 1 [file ijms-27-05293-s001.zip › ijms-4254549-supplementary.pdf]

SUPPLEMENTARY FIGURE S1

Figure S1. Correlation between HER2DX scores and HER2 gene copy number (FISH).

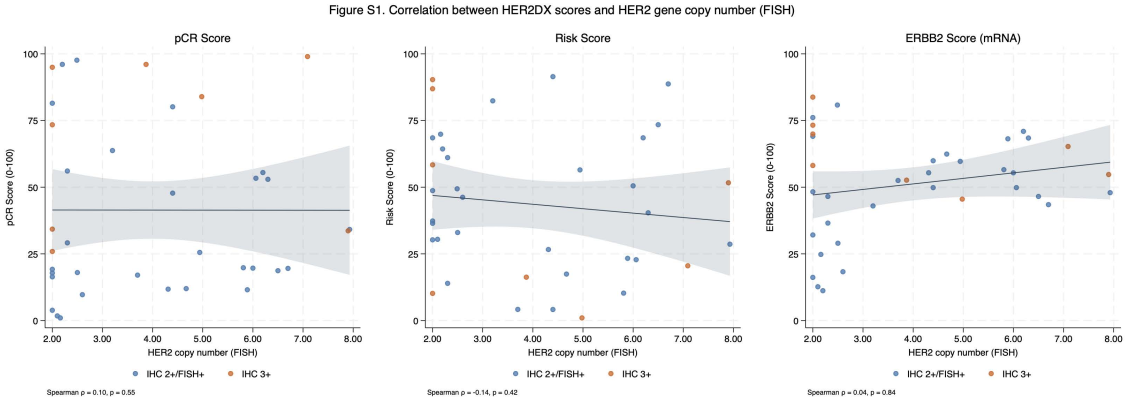

Correlation between HER2DX scores and HER2 gene copy number (FISH). Scatter plots show the association between each HER2DX score (pCR, Risk, and ERBB2 mRNA scores; continuous scale 0–100) and absolute HER2 signal count by dual-probe FISH in the 37 patients with available data. FISH was predominantly performed in IHC 2+ equivocal tumors per ASCO/CAP guidelines. Blue = IHC 2+/FISH+; orange = IHC 3+. Solid lines = linear regression; shaded areas = 95% CI. None of the scores correlated significantly with HER2 copy number (pCR score: $\rho = 0.10$, $p = 0.55$; Risk score: $\rho = -0.14$, $p = 0.42$; ERBB2 score: $\rho = 0.04$, $p = 0.84$), consistent with the concept that transcriptional HER2 output does not necessarily parallel genomic amplification.
